# Pilonidal Disease Mimicking Fistula-in-Ano in a 15-Year-Old Female

**DOI:** 10.1155/2012/310187

**Published:** 2012-09-09

**Authors:** Corey W. Iqbal, Alessandra C. Gasior, Charles L. Snyder

**Affiliations:** Department of Surgery, Children's Mercy Hospital and Clinics, 2401 Gillham Road, Kansas City, MO 64108, USA

## Abstract

Pilonidal disease typically presents with an abscess or intermittent pain and drainage in the sacrococcygeal region during the pubertal years. Further examination typically reveals pits in the midline of the sacrococcyx area due to entrapment of hair with recurrent entrapment, infection, and drainage. The following paper describes an unusual presentation of a pilonidal cyst with fissure and perianal drainage.

## 1. Introduction

Pilonidal disease was first described by O. H. Mayo in 1833 although the term pilonidal would be coined later by R. M. Hodges in 1880 [1]. It typically occurs in males between the ages of 15–30 years [2]. Patients may present acutely with an abscess in the sacrococcygeal region or with more chronic symptoms including intermittent pain and drainage in this same region. The following paper represents an unusual presentation of pilonidal disease with anal fissure and perineal drainage.

## 2. Case Report

An otherwise healthy 15-year-old female was referred to the surgery clinic with a chief complaint of perirectal irritation and drainage for 3 months. Her primary care physician had initially evaluated her and treated a presumed perianal abscess with oral antibiotics. Her acute symptoms resolved; however she had persistent irritation associated with intermittent drainage from her perianal region. Physical examination revealed a minimally tender external perianal opening with a small amount of purulent drainage. It was decided to perform an examination under anesthesia, with possible anal fistulectomy.

**Figure 1:**
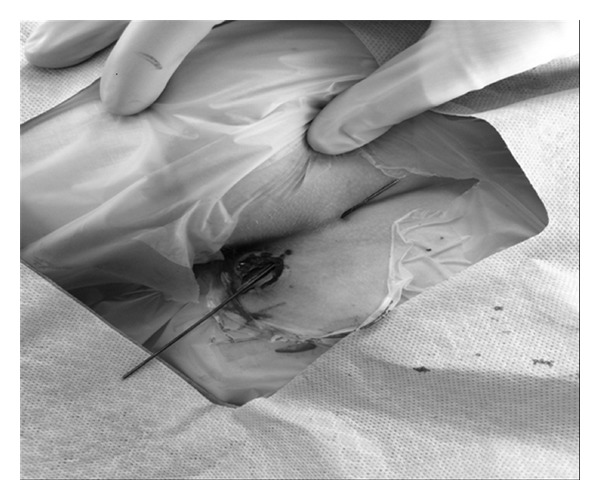


 On examination under anesthesia she had an external perianal opening at the 8 o'clock position, 2 cm from the anal verge with a small amount of mucous drainage. On anoscopy, there was no obvious internal opening. A chord was palpable tracking posteriorly towards the natal cleft. The external opening was cannulated using an angiocatheter and hydrogen peroxide with methylene blue was infused to identify any communication. On injection, blue dye or peroxide was not observed within the rectum, but blue dye and peroxide solution was identified from a small pit in the natal cleft. A lacrimal probe was then placed across the fistula tract which did not involve the anal sphincter. A fistulotomy was performed and a chronic pilonidal cavity was encountered (see Figure 1). A large amount of hair and granulation tissue was debrided and the pilonidal cavity was excised entirely without extending the fistulotomy tract to the midline to avoid an open wound in this location. The fistula tract was debrided and marsupialized with absorbable sutures. 

 The patient was followed postoperatively in the surgery clinic. At 2 months follow-up she was completely asymptomatic, and her wounds had completely healed without complication. 

## 3. Discussion

 Pilonidal cysts typically present with abscess or recurrent pain and drainage of the sacrococcygeal region. Physical examination usually reveals pits in the midline of this area. These pits are the result of the entrapment of foreign material (usually hair) with subsequent infection, suppuration, drainage, and sinus tract formation [3]. Various treatment strategies for managing pilonidal disease have been employed. Most of these strategies focus on keeping the incision off the midline (where the wound is at risk for breakdown) and creating a shallower cleft. It is recommended to initially approach pilonidal disease with less-extensive procedures, reserving more complicated approaches for patients who develop chronic pilonidal disease. Patients who present acutely with an abscess are often treated with an elliptical incision made lateral to the midline which incorporates all of the diseased tissue (including sinus tracts) and allows healing by secondary intention. The recurrence rate is 20% in this setting [4].

 Chronic pilonidal disease is associated with sinus tract formation which is almost exclusively limited to the sacrococcygeal region. The principals of management in this setting require excision of the sinuses and associated tracts. This can be done through incision and curettage of the tracts with or without marsupialization with recurrence rates of up to 19% [4]. This approach requires local wound care as the wound heals by secondary intention. Wide local excision with primary closure is usually associated with more wound complications and reported recurrence rates of 11–29% [4]. This dissection is taken down to the sacral fascia which likely contributes to the high incidence of wound complications by creating (or preserving) a deep cleft. Karydakis modified radical excision by proposing a curvilinear incision lateral to the midline with the creation of a gluteal flap and debridement of the sinus tracts [5]. This allows creation of a shallower cleft and keeps the wound off of the midline. Reported recurrence rates with this technique are less than 5% [6–8]. More extensive reconstruction procedures have been described as well including the rhomboid flap, V-Y flaps, and Z-plasty [9]. These techniques are typically reserved for patients with multiple recurrences or nonhealing wounds. Due to the extensive tissue mobilization, when these techniques fail they can result in large wounds. Recurrence rates are less than 5% when successful [9]. A recent Cochrane database analysis of the surgical management of pilonidal disease concluded: “It may well be that off-midline closure is superior, but given the impression of the results and the lack of similarity across trials for some of the outcomes, better evidence is needed before suggesting it become standard of care.” [10].

 Pilonidal disease with fistula and drainage around the anus represents an atypical presentation that is more likely to raise a suspicion of fistula-in-ano rather than pilonidal disease. Four cases were initially reported in 1948 [11]. A literature review found an additional 19 reported cases of pilonidal disease associated with a perianal fistula; however, we suspect that it is more prevalent than our literature search would reflect [12–14]. Previous reports identified a marked male predominance with a mean age at presentation of approximately 25 years. Our patient appeared to have secondary perianal disease, with the origin at the natal cleft (however, the natal cleft involvement was minimal) and extension to the perianal area. The fistulous connection can be superficial as in this case or deeper with sphincter penetration. Primary perianal pilonidal disease is quite rare and can be treated with local excision with or without primary closure [12, 14].

Pilonidal disease affecting a variety of other sites has also been reported including the perineum, interdigital spaces (i.e., hairdressers, suggesting an acquired etiology), umbilical, peritoneal catheter exit site, scalp, lateral buttock, and male and female genital tracts [15–23]. Pilonidal disease itself has an incidence of approximately 1 in 4,000 [2]. 

 In this subset of patients, the same principles for treating pilonidal disease must be employed in addition to obliteration of the fistula tract and preservation of the anal sphincter (although involvement of the sphincter by the fistula tract appears to be uncommon). Patients presenting with a perianal fistula should undergo a complete examination to identify an abscess, fistula-in-ano, or pilonidal disease. This can be achieved by an examination under anesthesia or the use of magnetic resonance imaging (MRI); the latter has become a useful adjunct in defining complex perianal disease and fistulous communications [1].

 We feel that a minimalist approach should be taken initially, reserving more complex techniques for recurrences. Since the fistula tract in our patient did not involve the anal sphincter, we managed the fistula tract with fistulotomy and marsupialization allowing it to heal by secondary intention. By opening the fistula tract, this provided a lateral approach to the pilonidal cavity and sinus which was excised avoiding a midline wound and our patient has done well. The largest series of pilonidal disease with perianal fistula in the literature described using fistulectomy in combination with a Karydakis flap and reported no recurrences with that approach as well [13].

## References

[1] Taylor SA, Halligan S, Bartram CI (2003). Pilonidal sinus disease: MR imaging distinction from fistula in ano. *Radiology*.

[2] Bascom J (2008). Surgical treatment of pilonidal disease. *British Medical Journal*.

[3] Da Silva JH (2000). Pilonidal cyst: cause and treatment. *Diseases of the Colon and Rectum*.

[4] Sohn N, Martz J, Cameron JL (2004). Pilonidal disease. *Current Surgical Therapy*.

[5] Karydakis GE (1973). New approach to the problem of pilonidal sinus. *The Lancet*.

[6] Karydakis GE (1992). Easy and successful treatment of pilonidal sinus after explanation of its causative process. *Australian and New Zealand Journal of Surgery*.

[7] Kitchen PRB (1996). Pilonidal sinus: experience with the Karydakis flap. *British Journal of Surgery*.

[8] Can MF, Sevinc MM, Yilmaz M (2009). Comparison of Karydakis flap reconstruction versus primary midline closure in sacrococcygeal pilonidal disease: results of 200 military service members. *Surgery Today*.

[9] Horwood J, Hanratty D, Chandran P, Billings P (2012). Primary closure or rhomboid excision and Limberg flap for the management of primary sacrococcygeal pilonidal disease? A meta analysis of randomized controlled trials. *Colorectal Disease*.

[10] Brasel KJ, Gottesman L, Vasilevsky CA (2010). Meta-analysis comparing healing by primary closure and open healing after surgery for pilonidal sinus. *Journal of the American College of Surgeons*.

[11] SMITH TE (1948). Anterior or perineal pilonidal cysts. *The Journal of the American Medical Association*.

[12] Vallance S (1982). Pilonidal fistulas mimicking fistulas-in-ano. *British Journal of Surgery*.

[13] Kulacoglu H, Dener C, Tumer H, Aktimur R (2006). Total subcutaneous fistulectomy combined with Karydakis flap for sacrococcygeal pilonidal disease with secondary perianal opening. *Colorectal Disease*.

[14] Taylor BA, Hughes LE (1984). Circumferential perianal pilonidal sinuses. *Diseases of the Colon and Rectum*.

[15] Vergles D, Cupurdija K, Lemac D, Legac A, Kopljar M (2010). Interdigital pilonidal sinus in a female hairdresser. *ANZ Journal of Surgery*.

[16] O’Neill AC, Purcell EM, Regan PJ (2009). Interdigital pilonidal sinus of the foot. *Foot*.

[17] Uysal AC, Alagöz MS, Ünlü RE, Şensöz O (2003). Hair dresser’s syndrome: a case report of an interdigital pilonidal sinus and review of the literature. *Dermatologic Surgery*.

[18] Dixit SP (1976). Pilonidal sinus of the umbilicus. *Canadian Journal of Surgery*.

[19] Naraynsingh V, Hariharan S, Dan D (2009). Umbilical pilonidal sinus: a new treatment technique of sinus excision with umbilical preservation. *Dermatologic Surgery*.

[20] Chiu DYY, Coward RA, Woywodt A, Gharpuray-Pandit D, Hart RO (2010). Development of pilonidal sinus in an old exit site four years after removal of the tenckhoff catheter. *Peritoneal Dialysis International*.

[21] Chiu MW, Abrishami P, Sadeghi P (2008). Letter: pilonidal cyst of the scalp. *Dermatologic Surgery*.

[22] Ikwueke I, Bandara S, Fishman SJ, Vargas SO (2008). Congenital dermal sinus tract in the lateral buttock: unusual presentation of a typically midline lesion. *Journal of Pediatric Surgery*.

[23] Baker T, Barclay D, Ballard C (2008). Pilonidal cyst involving the clitoris: a case report. *Journal of Lower Genital Tract Disease*.

